# Transcriptional responses to temperature and low oxygen stress in Atlantic salmon studied with next-generation sequencing technology

**DOI:** 10.1186/1471-2164-14-817

**Published:** 2013-11-22

**Authors:** Pål A Olsvik, Vibeke Vikeså, Kai K Lie, Ernst M Hevrøy

**Affiliations:** National Institute of Nutrition and Seafood Research, Nordnesboder 1-2, N-5005 Bergen, Norway; Skretting Aquaculture Research Center, P.O. Box 48N-4001, Stavanger, Norway

**Keywords:** Farmed salmon, Climate change, Temperature and hypoxia stress, Next-generation sequencing

## Abstract

**Background:**

Warmer seawater as a result of climate change may impose environmental challenges for Atlantic salmon aquaculture in its southernmost geographic range. Seawater temperatures above optimal level for growth may be reached in the warmest summer weeks. Caged fish can experience temperature and low oxygen saturation stress during such episodes, raising fish welfare and productivity concerns. In this work we compare the transcriptional responses in Atlantic salmon exposed to chronic high temperature (19°C) and low oxygen saturation (4-5 mg/L) stress.

**Results:**

We used next-generation sequencing and RT-qPCR to screen for effects, and focused on growth regulation and oxidative stress in fish exposed to sub-optimal conditions. Both prolonged temperature (45 days) and low oxygen (120 days) stress had a significant negative effect on growth. The main effect of heat stress appears to be a general reduced transcriptional rate in salmon liver, while mechanisms typically associated with responses induced by chemical drugs were stimulated. Heat stress significantly down-regulated several transcripts encoding proteins involved in the protection against oxidative stress, including CuZn SOD, Mn SOD, GPx1 and GR, as well as additional stress markers HIF1A, CYP1A, MTOR and PSMC2 (RT-qPCR data). In salmon held at low oxygen concentration for four months protein ubiquitination (protein catabolism) was the most strongly affected pathway. According to the RT-qPCR data, low oxygen stress significantly up-regulated the transcriptional levels of IGFBP1B and down-regulated the levels of GR. Pathway analysis suggests that high temperature and low oxygen saturation stress affects many similar mechanisms in Atlantic salmon. Based on the gene lists, six out of the top ten predicted upstream transcriptional regulators, 1,2-dithiol-3-thione sirolimus, CD437, 5-fluorouracil, HNF4A and NFE2L2, were similar between the two treatments.

**Conclusions:**

In conclusion, temperature and low oxygen saturation stress affect many identical mechanisms in liver cells resulting in a metabolic depression, but these effects are not necessarily mediated through altered transcription of the same genes.

**Electronic supplementary material:**

The online version of this article (doi:10.1186/1471-2164-14-817) contains supplementary material, which is available to authorized users.

## Background

Climate change may introduce several environmental challenges for farmed fish caged in seawater pens. In temperate areas, increased seawater temperature during the summer months may represent a problem for farmed fish unable to swim to colder water. Ectothermic animals such as fish normally show temperature-dependent oxygen consumption [1]. Increasing temperatures may induce low oxygen stress since oxygen solubility is reduced in warmer water. Fish may also experience hypoxia at elevated temperatures even under conditions with unchanged oxygen tension, for example increased temperature could reduce the binding capacity of hemoglobin for oxygen transport [2, 3]. Environmental temperature affects almost all aspects of fish physiology, and effects of temperature fluctuation on teleosts have been studied extensively for decades [4]. Sedentary fish species may be exposed to stressful short-term temperature spikes during the summer months when ambient seawater temperature changes rapidly. Metabolism in ectothermic fish is highly dependent on environmental temperature [5], with suboptimal conditions affecting both feed intake and growth [6]. For instance, sea-caged Atlantic salmon (*Salmo salar*), unable to escape sudden temperature bursts by vertical migration, may experience considerable challenges with temperature adaptation. In southern Norwegian fjords such temperature periods lasting a few weeks have been recorded in recent years with observed temperatures above optimal levels for locally farmed Atlantic salmon [6], raising both fish welfare and productivity challenges.

Numerous studies have profiled global gene expression changes in fishes exposed to elevated temperature and hypoxia. Both acute and chronic heat stress can induce relative large transcriptional changes in salmonids [2, 4, 7, 8] and other fish species [3, 9–11], including in cold-adapted Antarctic fish [12, 13]. Also hypothermia can induce transcriptional changes in fishes [14, 15]. Depending on studied cell type, typical responses in fish exposed to heat stress include altered transcription in genes involved in protein processing, transcription and cell growth [9], protein folding and heat shock proteins [2, 8, 10, 11], cell cycle arrest and apoptosis, and proteolytic protein degradation [11], β-oxidation of fatty acids and peroxisome proliferator-activated receptor genes [12, 13], acute inflammatory response [12], and ribosomal genes and catabolism, i.e. metabolism [7, 16]. Although oxidative stress response has been reported as one of the strongest affected mechanisms in fish exposed to acute temperature stress according to global genome screening [12], surprisingly few of these studies have reported large transcriptional changes in genes associated with the antioxidative defense.

As for temperature, hypoxia may have profound effect on biochemical, molecular and physiological processes in fish [17]. Consequences of low dissolved oxygen are often sublethal and affect growth, immune responses and reproduction [18]. For Atlantic salmon, oxygen levels falling below the critical point of 6 mg/L are considering hypoxic [19]. In one of the first microarray screening studies with fish, Gracey et al. [20] showed profound effects of hypoxia on the longjawed mudsucker (*Gillichthys mirabilis*) after 5 days of treatment. They observed effects on transcripts encoding proteins involved in protein synthesis and locomotion, as well as on genes needed for anaerobic ATP production. Studying gene expression changes in zebrafish (*Danio rerio*) embryos exposed to 24 hours of hypoxia during development, Ton et al. [21] identified transcriptional changes indicating metabolic depression with a switch from aerobic to anaerobic metabolism and energy preservation. More recent studies have shown changes in expression of genes related to physiological adaptation to low environmental oxygen in gills of zebrafish after 21 days of hypoxia exposure [22], effects on general metabolism, catabolism, and ubiquitin-proteasome pathway in brain, gill and liver of Japanese medaka (*Oryzias latipes*) after 5-6 days of exposure to hypoxia [23, 24], responses in genes involved in metabolic energy changes in brain, gill and liver of *Xiphophorus maculatus* after 6 days of exposure to hypoxia [25], as well as activation of glycolysis and oxidative phosphorylation in fin tissue of Japanese medaka after 7 days of exposure to hypoxia [26]. In gonads of zebrafish acute hypoxia for 4 and 14 days affected responses such as metabolism of carbohydrate and proteins, and reactive oxygen species metabolism, while chronic hypoxia affected lipid metabolism, steroid hormones, and immune responses [27].

In this work we wanted to evaluate how Atlantic salmon cope with environmental challenges possibly imposed by climate change by comparing the molecular responses in fish held at sub-optimal temperature and oxygen levels. Recent findings suggest that thermal responses of fish can be oxygen-limited [28], indicating that oxygen-limited thermal tolerance is of major importance in the response of fish to climate change [29]. Global transcriptional responses in liver of adult Atlantic salmon exposed to high temperature (19°C) and low oxygen (4-5 mg O_2_/L) was examined using next-generation sequencing technology. Tissue samples were obtained from two independent experiments. In the first experiment, described in detail by Hevrøy et al. [30], Atlantic salmon was held at 13°C (optimal temperature, control), 15°C, 17°C or 19°C (heat stressed). Effects of heat stress were evaluated by comparing the transcriptional patterns in the 13°C and 19°C groups of fish using suppression subtractive hybridization (SSH) cDNA libraries followed by FLX 454 sequencing. In the second experiment, control fish were held at optimal oxygen (7-8 mg/L) and low oxygen stressed fish held at sub-optimal oxygen (4-5 mg/L). Effects of low oxygen were evaluated by comparing the transcriptional patterns in the two groups of fish using normalized cDNA libraries followed by FLX 454 sequencing. In total we sequenced about 1.5 million reads from four cDNA libraries, and used gene ontology and pathway analysis tools to evaluate the responses. Real-time RT-qPCR analysis was used on an extended number of fish for follow-up examination of relevant genes, including growth regulation and oxidative stress relevant genes.

## Results

### Temperature and low oxygen trials, growth

**Figure 1:**
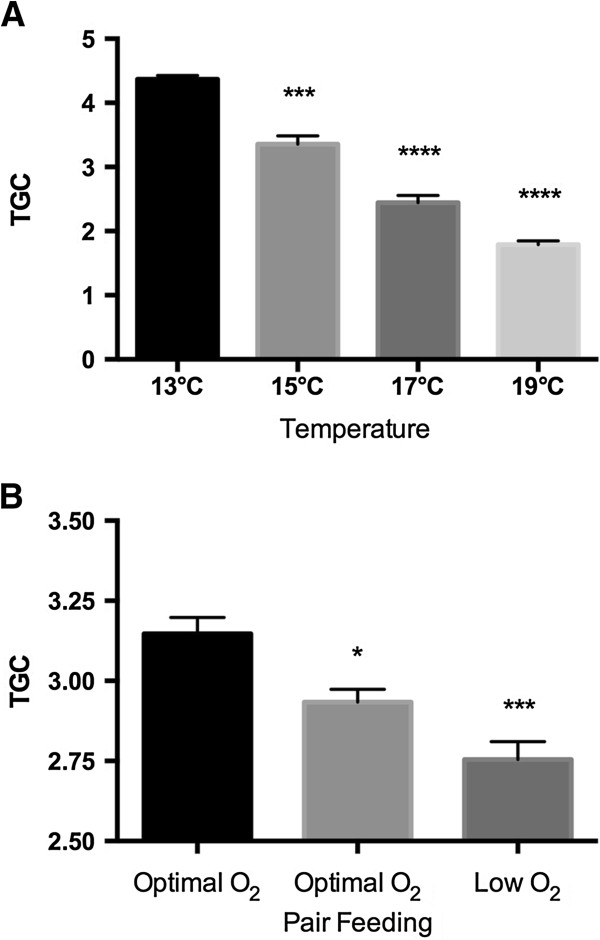
**Thermal growth coefficient (TGC) of Atlantic salmon exposed to A) high temperature and B) low oxygen stress.** Mean ± SEM. *P<0.05, ***P<0.001, ****P<0.0001.

Somatic growth of Atlantic salmon, measured as thermal growth coefficient (TGC), significantly decreased with increasing exposure temperature (1-way ANOVA, Figure 1A). In salmon exposed to low dissolved oxygen saturation there was a significant reduction in TGC compared to fish kept at normal oxygen saturation levels (1-way ANOVA, Figure 1B). Pair fed fish raised on normal oxygen saturation had reduced TGC compared to normal fed fish at kept at similar oxygen level, but higher growth than fish kept at low dissolved oxygen saturation (1-way ANOVA, Figure 1B). By comparing fish growth from these two studies, comparable TGC values (<3, Figures 1A and 1B) were observed in fish held at sub-optimal temperatures above 15°C with optimal oxygen, in pair fed fish kept at with optimal oxygen levels, and in fish exposed to low dissolved oxygen saturation.

### 454 FLX sequencing and assembly

Table 1 shows an overview of the 454 FLX sequencing data. In total, 1,425,167 reads were sequenced from the four cDNA libraries. A total of 773,725 sequence reads, with an average read length of 333 nucleotides, were obtained from the temperature stress experiment (forward and reverse SSH cDNA libraries), while 651,442 sequence reads with an average read length of 526 nucleotides were obtained from the low oxygen experiment (normal O_2_ and low O_2_ normalized cDNA libraries). The number of reads sequenced from the forward and reverse SSH cDNA libraries were 313 713 and 427 812, respectively. The total number of reads sequenced from the normal oxygen cDNA library was 357 347, while 401 270 reads were sequenced from the low oxygen cDNA library.

**Table 1:** **Summary of 454 sequencing data**

	Temperature		Hypoxia	
	13°C (EH1)	19°C (EH2)	Normal O_2_	Low O_2_
Tissue	Liver	Liver	Liver	Liver
Individuals	6	6	9	9
Method	SSH library	SSH library	Normalized library	Normalized library
Sequencing	FLX 454 GS-titanium	FLX 454 GS-titanium	FLX 454 GS-titanium	FLX 454 GS-titanium
Platform	CEES, UiO	CEES, UiO	LGC Genomics, Munich	LGC Genomics, Munich
Reads	334403	439322	375347	401270
All contigs	14325	5659	10827	11478
Large contigs	2289	1790	7817	8498
IPA input >50 reads	754	1229	1466	1591

Assembly of sequenced reads from the SSH cDNA libraries was conducted with the GS De Novo Assembler 2.6 (Newbler software, build 20110523_1851). Using a minimum overlap length of 40 and minimum overlap identity of 90, 186 508 reads (59.5%) were assembled from the forward SSH cDNA library (up-regulated by 19°C). The number of partially assembled reads from this library was 26 831, and the number of singletons was 37 564. The total number of contigs from the forward SSH cDNA library was 5 067. Of these, 1 928, with an average length of 664 bp, were large contigs (>500 bp). From the reverse SSH cDNA library (down-regulated by 19°C) 303 804 reads were assembled (71.0%). 56 730 reads from this library were partially assembled and the cDNA library contained 43 432 singletons. The total number of contigs obtained from the reverse SSH cDNA library was 5 659, while the number of large contigs with a length of >500 bp was 1 790. These had an average length of 626 bp.

Using Newbler 2.6 (build 20110517_1502) with GS-Assembler parameters with minimum overlap length of 40 and a minimum overlap identity of 90, 264 911 reads were assembled from the normal oxygen cDNA library. The number of partial assembled reads from this cDNA library was 26 667, while the number of singletons was 57 604. In total, 10 827 contigs were obtained from the normal oxygen cDNA library, of which 7 817 (>500 pb) with an average length of 1 173 bp were used for downstream analyses. From the low oxygen cDNA library, 283 599 reads were assembled. From this cDNA library, the number of partially assembled reads was 28 095 while the number of singletons was 58 462. The total number of contigs from this cDNA library was 11 478, of which 8 498 were large contigs (>500 bp). Large contigs from the low oxygen cDNA library had an average size of 1 191 bp.

### Annotation and gene ontology (GO) enrichment analysis

To search for possible impurities in the cDNA pool, MEGAN software was used. Metatranscriptomic analysis showed that reads from all four cDNA libraries had very few hits against microbial and human sequences, and with a vast majority of hits against sequences from teleostean species, suggesting a very low level of contamination in the cDNA pools.

Of the 754 contigs consisting of more than 50 reads from the forward SSH cDNA library (19°C group), 58.0% were annotated with a Blastx cut-off of 10-6 (Additional file 1A). 28.1% of the contigs showed no Blastx hits against the GenBank database. From the reverse SSH cDNA library (13°C group), 51.7% of the 1229 contigs consisting of more than 50 reads were annotated with a Blastx cut-off of 10-6 (Additional file 1B). 36.3% of the contigs from the reverse SSH cDNA library showed no Blastx hits. Similarity distribution showed most hits against Atlantic salmon sequences. Of the 1591 contigs consisting of more than 50 reads from the low oxygen stress normalized cDNA library (Additional file 2A), 85.8% were annotated with a Blastx cut-off of 10-6. 7.5% of the contigs showed no Blastx hits against the GenBank database. From the normal oxygen normalized cDNA library, 85.3% of the 1466 contigs consisting of more than 50 reads were annotated with a Blastx cut-off of 10-6 (Additional file 2B). 8.0% of the contigs from the normal oxygen normalized cDNA library showed no Blastx hits. Similarity distribution of the SSH cDNA contigs showed most hits against Atlantic salmon sequences, while similarity distribution of the normalized cDNA contigs showed most hits against zebrafish sequences, closely followed by Atlantic salmon sequences.

**Figure 2:**
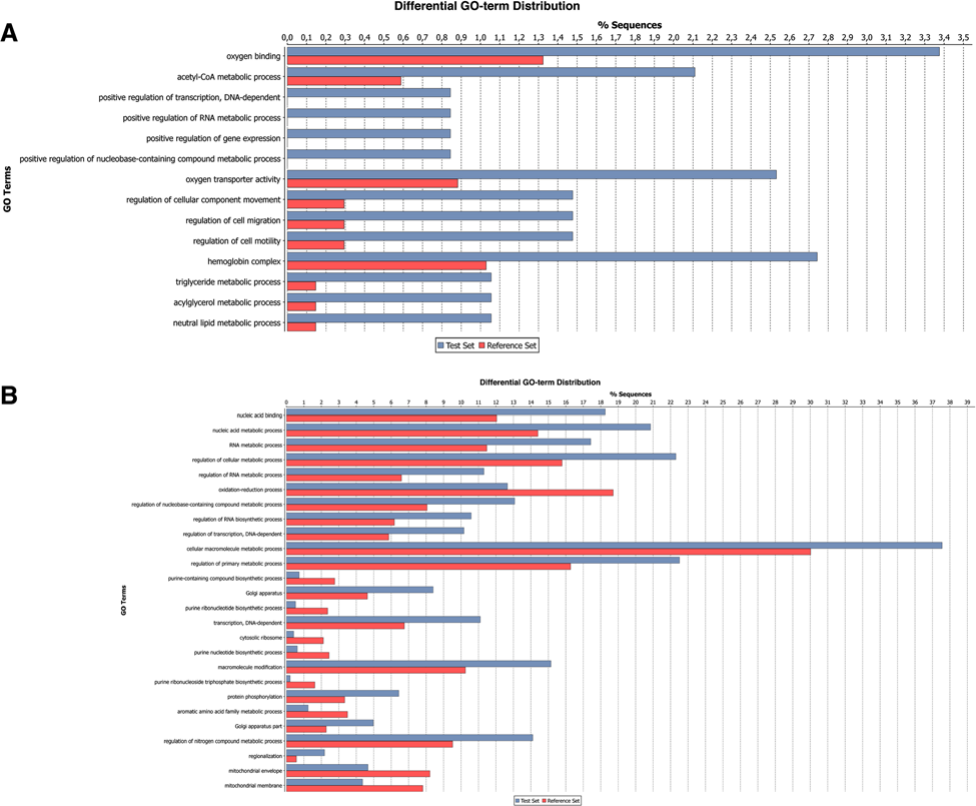
**Enriched GOs in Atlantic salmon exposed to high temperature or low oxygen concentration.** The data were obtained with Fisher’s Exact test as implemented in Blast2GO. **A)** Temperature SSH cDNA libraries. **B)** Low versus normal oxygen cDNA libraries.

GO enrichment analysis was performed by using the Fisher’s Exact Test, as implemented in the Blast2GO software, to study temperature- or low oxygen- specific responses in Atlantic salmon using contigs containing more than 50 reads. Figure 2 shows over- or under expressed GOs as determined by the Fisher’s Exact Test (P < 0.05). Figure 2A shows enriched GOs in Atlantic salmon exposed to 19°C. Relative few GOs were differentially expressed in heat stressed fish at 19°C compared to fish kept at optimal temperature at 13°C. Heat stress resulted in overexpression of GOs linked to oxygen binding and transporter activity, i.e. GO:0019825 *oxygen binding* and GO:000534 *oxygen transporter activity*. Figure 2B shows differentially expressed GOs in Atlantic salmon exposed to low oxygen saturation with significance levels of P < 0.001. Many GOs linked to general metabolism were significantly affected by low oxygen saturation, suggesting a compensatory response induced by low oxygen stress. Top listed overexpressed GOs were GO:0090304 *nucleic acid metabolic process*, GO:0016070 *RNA metabolic process* and GO:0031323 *regulation of cellular metabolic process*, whereas GOs linked to oxygen reduction reactions such as GO:0055114 *oxidation-reduction process* and GO:0016491 *oxidoreductase activity* were underexpressed. All significant over- or underexpressed GOs, including IDs and terms, P-values, and the number of transcripts associated with a specific GO term from the cDNA libraries, as determined by Fisher’s Exact Test, are shown in Additional file 3. From the temperature stress experiment, enriched GOs are shown in the Additional file 3, worksheet A. Form the low oxygen experiment, we list GO enrichment analyses data both for contigs only consisting of more than 50 reads (>50 reads) (worksheet B) and by using all large contigs (worksheet C). According to the latter analysis, GOs linked to lipid metabolism were underexpressed in salmon held at low oxygen saturation, with GO:0006629 *lipid metabolic process* and GO:0006631 *fatty acid metabolic process* being most significant. In total, by also including contigs with less than 50 reads, fewer significant GOs were found (133 versus 266). The larger dataset however appears to provide more specific GOs than by using only contigs consisting of >50 reads.

### Functional analyses

**Figure 3:**
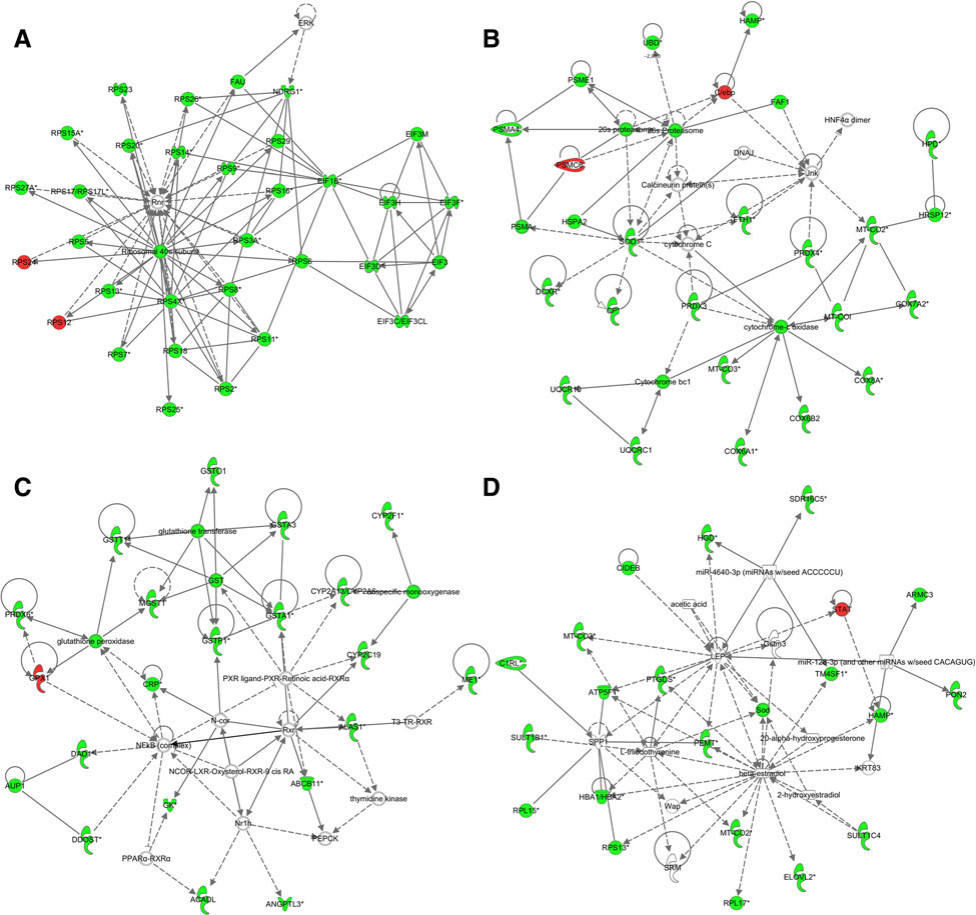
**Biological networks derived using IPA network analysis from the temperature stress experiment.** The top networks **A)** “*Protein Synthesis, Gene Expression, Cancer*” (rank 1, score 53), **B)** “*Hematological Disease, Metabolic Disease, Nutritional Disease*” (rank 4, score 36), **C)** “*Drug Metabolism, Protein Synthesis, Glutathione Depletion In Liver*” (rank 6, score 30), and **D)** “*Free Radical Scavenging, Lipid Metabolism, Molecular Transport*” (rank 7, score 28) are shown. Based on 1066 gene identifiers.

A gene list consisting of 1066 identifiers recognized by IPA from the temperature stress experiment was used for pathway analysis. The list was generated by considering all the genes in the forward SSH cDNA library as being up-regulated by temperature stress and given a fold-change score of 2, and all the genes in the reverse SSH cDNA library as being down-regulated by temperature stress and given a fold-change score of -2. IPA analysis for temperature stress genes revealed seven top networks, “*Protein Synthesis, Gene Expression, Cancer*” (score 53) (Figure 3A), “*RNA Post-Transcriptional Modification, Gene Expression, Protein Synthesis*” (score 48), “*DNA Replication, Recombination, and Repair, Energy Production, Nucleic Acid Metabolism*” (score 37), “*Hematological Disease, Metabolic Disease, Nutritional Disease*” (score 36) (Figure 3B), “*Gene Expression, Protein Synthesis, Cell-To-Cell Signaling and Interaction*” (score 36), “*Drug Metabolism, Protein Synthesis, Glutathione Depletion In Liver*” (score 30) (Figure 3C), and “*Free Radical Scavenging, Lipid Metabolism, Molecular Transport*” (rank 7, score 28) (Figure 3D). Top canonical pathways were “*EIF2 Signaling*” (P-value 1,03E-55, ratio 62/200 (0,31)), “*Acute Phase Response Signaling*” (P-value 1,11E-22, ratio 34/179 (0,19)), “*Regulation of eIF4 and p70S6K Signaling*” (P-value 5,74E-20, ratio 30/17 5 (0,171)), “*mTOR Signaling*” (P-value 1,32E-17, ratio 31/211 (0,147)), and “*Mitochondrial Dysfunction*” (P-value 9,3E-17, ratio 28/186 (0,151)). IPA Upstream Regulator analytic aim to identify the cascade of upstream transcriptional regulators that can explain the observed gene expression changes in the dataset, and help illuminate the biological activities occurring in the tissues or cells being studied. IPA analysis suggested that “*1,2-dithiol-3-thione*” (P-value 6,49E-08), “*sirolimus*” (P-value 2,50E-07), “*pirinixic acid*” (P-value 2,80E-07), “*CD 437*” (P-value 3,64E-07), and “*5-fluorouracil*” (P-value 3,93E-06) to be the top upstream regulators. Additional file 4 shows the predicted upstream regulators of temperature stress in Atlantic salmon liver with an overlap P-value higher than 10E-05 (worksheet “Temperature”). IPA-Tox analysis generates a focused toxicity and safety assessment of chemical compounds using toxicogenomics approaches, but can also be used to assess molecular perturbation of all kinds of environmental stressors, i.e. temperature and low oxygen. From the temperature stress experiment IPA-Tox analysis listed “*Mitochondrial Dysfunction*” (P-value 4,96E-15, ratio 25/150 (0,167)), “*Positive Acute Phase Response Proteins*” (P-value 2,78E-14, ratio 13/30 (0,433)), “*LXR/RXR Activation*” (P-value 5,83E-14, ratio 22/124 (0,177)), “*Negative Acute Phase Response Proteins*” (P-value 6,33E-14, ratio 8/8 (1)) and “*LPS/IL-1 Mediated Inhibition of RXR Function*” (P-value 4,11E-10, ratio 25/247 (0,101)) as the top five most significant effects.

**Figure 4:**
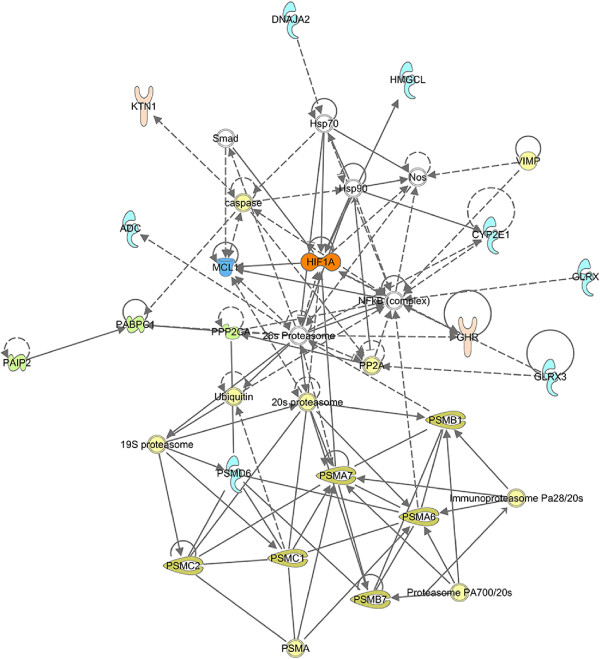
**Biological network derived using IPA network analysis from the low oxygen stress experiment.** The top network “*Nucleic acid metabolism, small molecule biochemistry, organ morphology*” (rank 1, score 33) is shown. Based on 221 gene identifiers.

The IPA Compare function was used to find transcripts solely expressed in the low oxygen group and not in the normal oxygen group, creating a list consisting of 221 genes with positive IPA identifiers. According to the IPA Core analysis, the top three affected networks solely expressed in the low oxygen group were “*Lipid Metabolism, Small Molecule Biochemistry, Dermatological Diseases and Conditions*” (score 77), “*Nucleic Acid Metabolism, Small Molecule Biochemistry, Organ Morphology*” (score 33) and “*Hereditary Disorder, Metabolic Disease, Cardiovascular Disease*” (score 33). Figure 4 shows the second highest scored IPA Core Analysis network by using gene identifiers unique for the low oxygen group. This network, “*Nucleic acid metabolism, small molecule biochemistry, organ morphology*”, includes the *hif1a* gene in a central position, suggesting a link to hypoxia. The top canonical pathway, “*Protein Ubiquitination Pathway*”, had a P-value of 8,68E-05 and a ratio of 11/268 (0,041). According to the IPA Core analysis “*1,2-dithiol-3-thione*” (P-value 6,49E-08), “*sirolimus*” (P-value 2,50E-07), “*pirinixic acid*” (P-value 2,80E-07), “*CD 437*” (P-value 3,64E-07) and “*5-fluorouracil*” (P-value 3,93E-06) were the top upstream regulators. Additional file 4 lists the predicted upstream regulators of low oxygen stress in Atlantic salmon liver with an overlap P-value higher than 10E-03 (worksheet “Hypoxia”). Activation z-scores from the low oxygen experiment were not possible to calculate since we did not have any fold-change input. Low oxygen exposure induced the following effects according to the IPA-Tox analysis, “*Glutathione Depletion - Phase II Reactions*” (P-value 1,11E-03, ratio 3/20 (0,15)), “*Hypoxia-Inducible Factor Signaling*” (P-value 6,08E-03, ratio 4/70 (0,057)), *Cholesterol Biosynthesis* (P-value 1,17E-02, ratio 2/16 (0,125)), *Cytochrome P450 Panel - Substrate is a Xenobiotic* (Human) (P-value 1,64E-02, ratio 2/19 (0,105)) and *Mitochondrial Dysfunction* (P-value 2,03E-02, ratio 5/150 (0,033)).

**Figure 5:**
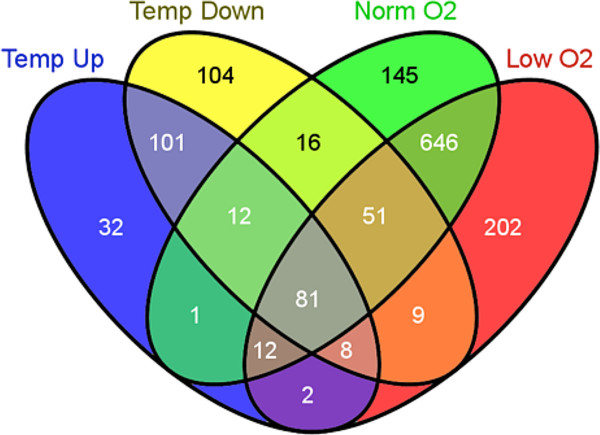
**Venn diagram displaying the number of significantly differentially expressed transcripts in Atlantic salmon exposed to temperature stress at 19°C (separated into up- and down-regulated transcripts) compared to fish kept at optimal temperature at 13°C.** Based on contigs assembled from more than 50 reads with IPA readable human identifiers.

To compare the degree of overlap in response to temperature and low oxygen stress and how these stressors differentially affect Atlantic salmon, the data were analyzed with the Venny tool [31]. Only 19 transcripts, listed in Table 2, were common for the two different stressors (Figure 5). In general, both treatments appear to have affected overall transcription and metabolism.

**Table 2:** **Function of common elements obtained from the high temperature stress experiment and the low oxygen stress experiment**

Gene symbol	Gene product	Temperature effect	Metabolic function	Protein function
ADK	Adenosine kinase	Up	ATP/ITP metabolism	ATP dependent phosphorylation of adenosine and other related nucleoside analogs to monophosphate derivatives
BTD	Biotinidase	Up	Biotin metabolism	Catalytic release of biotin from biocytin, the product of biotin-dependent carboxylases degradation
CREB3L3	cAMP responsive element binding protein 3-like 3	Up	Transcription regulation	Transcription factor that may act during endoplasmic reticulum stress by activating unfolded protein response target genes
CYP1A	Cytochrome P450, family 1, subfamily A	Up	Monooxygenase activity	Oxidization of a variety of structurally unrelated compounds, including steroids, fatty acids, and xenobiotics
GSTA1	Glutathione S-transferase alpha 1	Up	Glutathione metabolic process	Conjugation of reduced glutathione to a wide number of exogenous and endogenous hydrophobic electrophiles
KNG1	Kininogen 1	Up	Inflammatory response	Multiple function, high molecular weight kininogen (HMWK) involved in blood coagulation
RPL10	Ribosomal protein L10	Up	Translation	Component of the large 60S ribosomal subunit
RPL19	Ribosomal protein L19	Up	Translation	Component of the large 60S ribosomal subunit
RPL3	Ribosomal protein L3	Up	Translation	Component of the large 60S ribosomal subunit
TSTD1	Thiosulfate sulfurtransferase (rhodanese)-like domain containing 1	Up	Tumorigenesis?	Highly expressed in liver, possible role in tumorigenesis
BTF3	Basic transcription factor 3	Down	Regulation of transcription	Required for the initiation of transcription
CPN1	Carboxypeptidase N, polypeptide 1	Down	Proteolysis	Protects the body from potent vasoactive and inflammatory peptides released into the circulation
EIF3C/EIF3CL	Eukaryotic translation initiation factor 3, subunit C/-like	Down	Initiation of protein synthesis	Component of the eukaryotic translation initiation factor 3
H2AFV	H2A histone family, member V	Down	Nucleosome assembly	Play a central role in transcription regulation, DNA repair, DNA replication and chromosomal stability
HTRA1	High-temperature requirement A serine peptidase 1	Down	Regulation of cell growth/proteolysis	Serine protease with a variety of targets. Regulates the availability of insulin-like growth factors (IGFs)
NDUFS1	NADH dehydrogenase (ubiquinone) Fe-S protein 1, 75 kDa (NADH-coenzyme Q reductase)	Down	Mitochondrial electron transport, NADH to ubiquinone	Core subunit of the mitochondrial membrane respiratory chain NADH dehydrogenase
PABPC1	Poly(A) binding protein, cytoplasmic 1	Down	mRNA polyadenylation	May be involved in cytoplasmic regulatory processes of mRNA metabolism
PNP	Purine nucleoside phosphorylase	Down	Nucleobase-containing compound metabolic process	Catalyze phosphorolysis of purine nucleosides
PSAP	Prosaposin	Down	Lipid metabolic process	Catabolism of glycosphingolipids with short oligosaccharide groups

### RT-qPCR analyses

**Figure 6:**
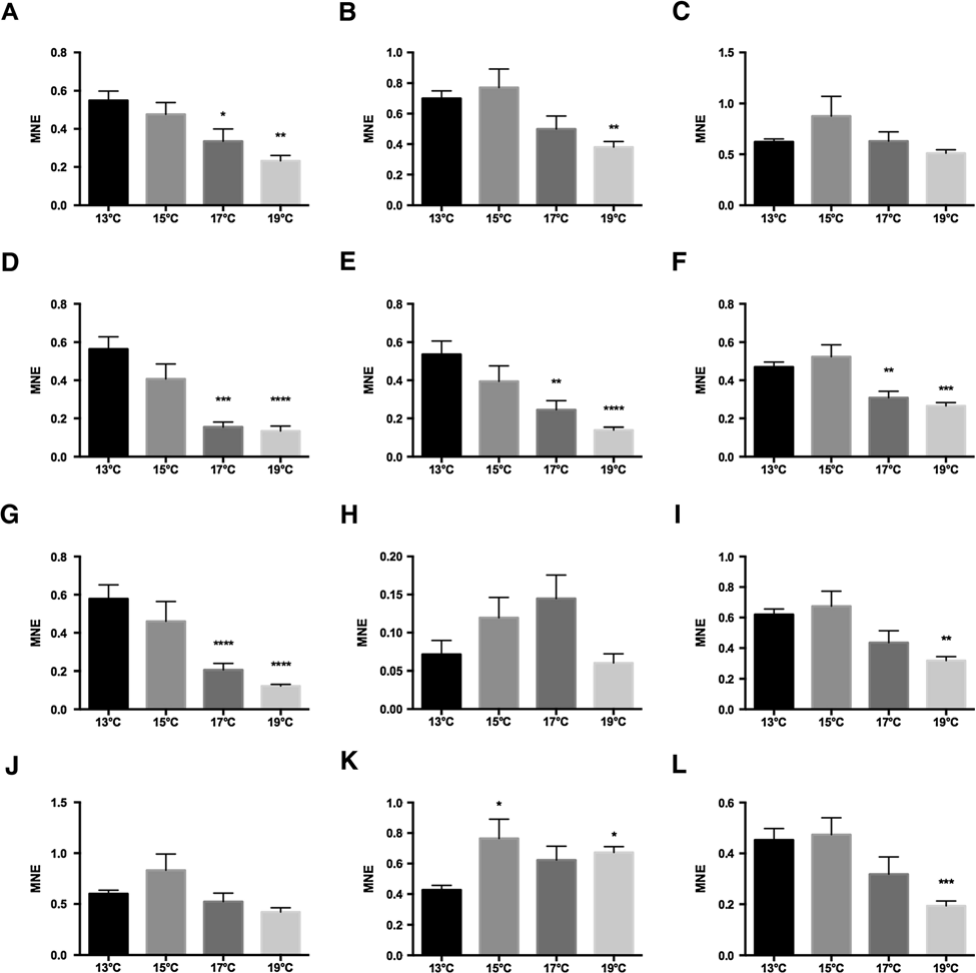
**Transcription of 12 genes determined with RT-qPCR in liver of adult Atlantic salmon kept at four different temperatures for 45 days. A)** CuZn SOD, **B)** Mn SOD, **C)** CAT, **D)** GPx1, **E)** GR, **F)** HIF1A, **G)** CYP1A, **H)** IGFBP1A, **I)** MTOR, **J)** NDUFS1, **K)** HSP90B and **L)** PSMC2. Each value represents the mean ± SEM (n = 9). Significant differences were identified with 1-way ANOVA analysis. *P < 0.05, **P < 0.01, ***P < 0.001. ****P < 0.0001. MNE = Mean Normalized Expression.

From the temperatures stress experiment, transcriptional levels of 12 target genes were determined with RT-qPCR in liver of adult salmon from 36 individual fish kept at four different temperatures (13°C, 15°C, 17°C and 19°C) for 45 days. Markers of oxidative stress (CuZn SOD, Mn SOD, CAT, GPx1 and GR), hypoxia (HIF1A), anti-growth/catabolism (IGFBP1A) and five genes (CYP1A, HSP90B, NDUFS1, MTOR and PSMC2) selected from the cDNA library gene lists (Figure 6) were included for analysis. CuZn SOD showed a decreasing expression with increasing temperature, and was significantly lower expressed in liver of fish kept at 17°C and 19°C compared to the control fish kept at 13°C (Figure 6A). Mn SOD was significantly lower expressed in fish kept at 19°C compared to the fish kept at 13°C (Figure 6B). Significant lower expression in fish kept at the two highest temperatures compared to the control fish was also observed for GPx1 (Figure 6D), GR (Figure 6E), HIF1A (Figure 6F) and CYP1A (Figure 6G). MTOR (Figure 6I) and PSMC2 (Figure 6L) expression was significantly lower in the fish kept at 19°C compared to the 13°C control, whereas no significant effects of temperature stress were observed between the groups for CAT (Figure 6C), IGFBP1A (Figure 6H) and NDUFS1 (Figure 6J). Significance levels are shown in the figures.

**Figure 7:**
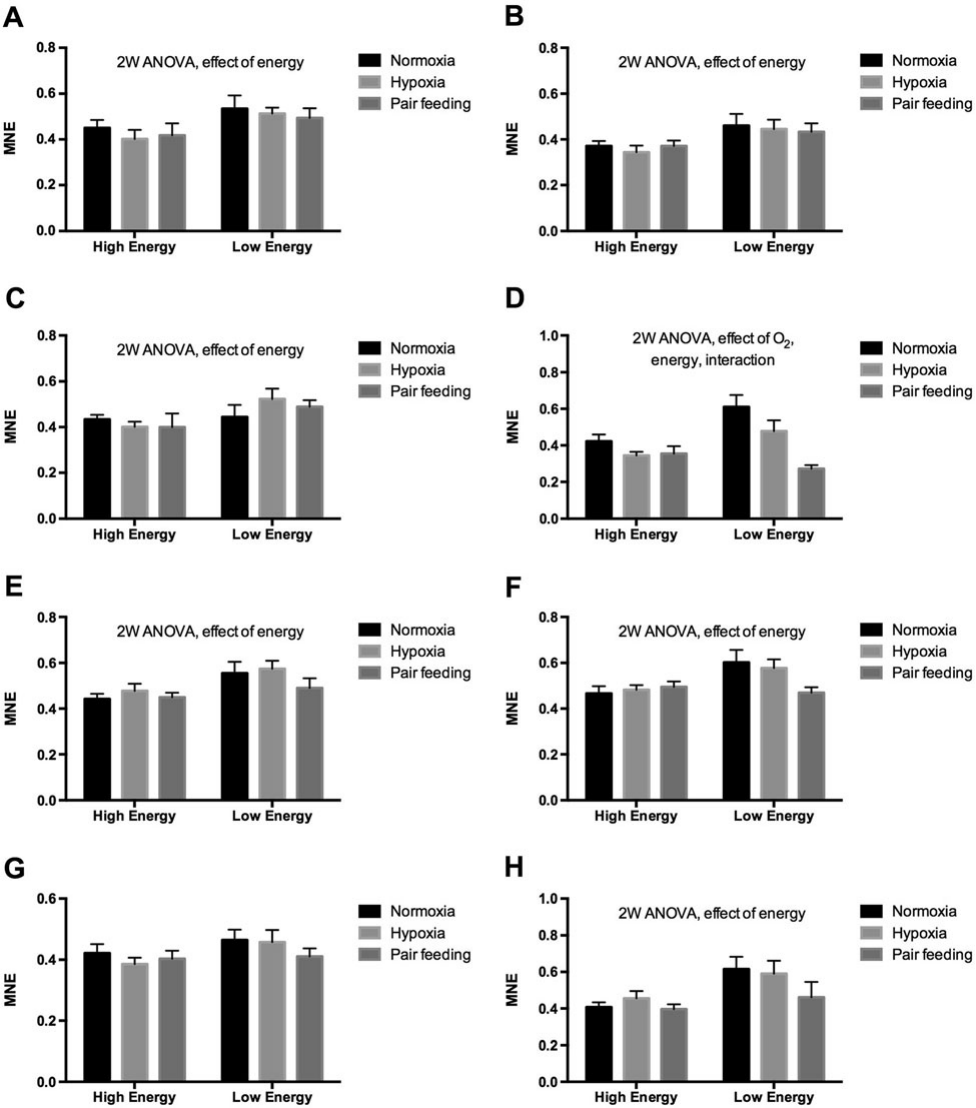
**Transcription of 12 genes determined with RT-qPCR from the low oxygen stress experiment.** The data were obtained from liver of Atlantic salmon kept at normal oxygen saturation (normoxia), kept at low oxygen saturation (hypoxia), or from fish pair fed on level with the hypoxia-exposed fish. **A)** CuZn SOD, **B)** Mn SOD, **C)** CAT, **D)** GR, **E)** HSP70, **F)** HSP90A, **G)** HIF1A and **H)** CYP1A. Each value represents the mean ± SEM (n = 9). Significant differences were identified with a 2-way ANOVA analysis. Significant effects of oxygen exposure levels, feed energy and interactions are shown in the figures. MNE = Mean Normalized Expression.

**Figure 8:**
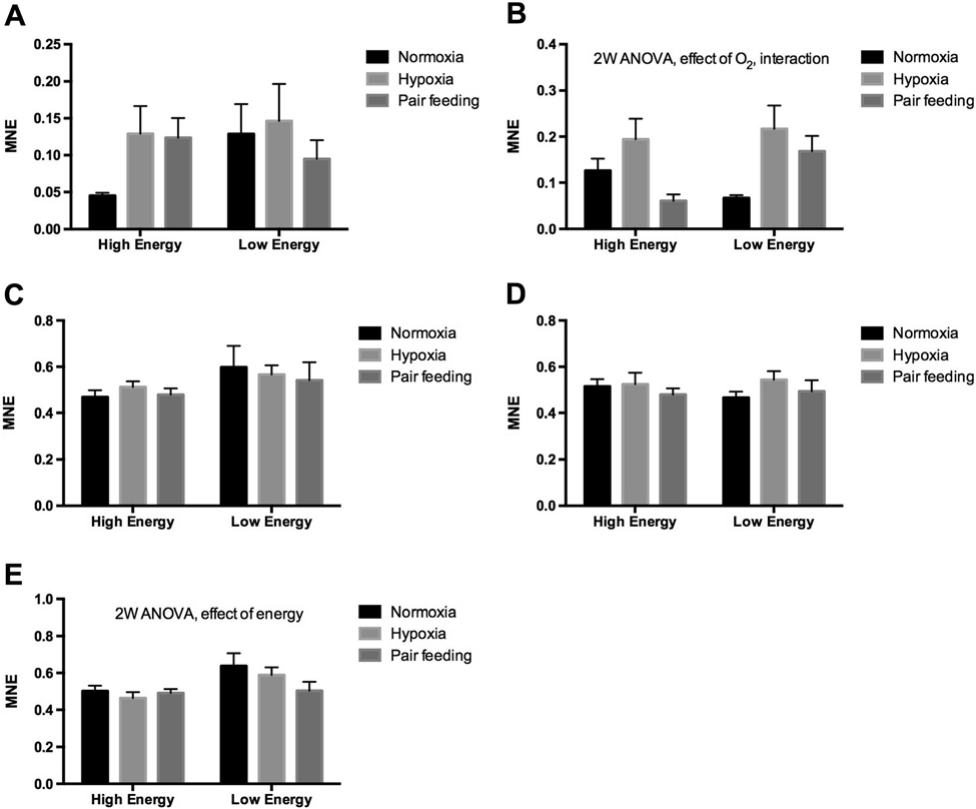
**Transcription of 12 genes determined with RT-qPCR from the low oxygen experiment.** The data were obtained from liver of Atlantic salmon kept at normal oxygen saturation (normoxia), kept at low oxygen saturation (hypoxia), or pair fed on level with the hypoxia-exposed fish. **A)** IGFBP1A, **B)** IGFBP1B, **C)** MTOR, **D)** NDUFS1 and **E)** PSMC2. Each value represents the mean ± SEM (n = 9). Significant differences were identified with a 2-way ANOVA analysis. Significant effects of oxygen exposure levels, feed energy and interactions are shown in the figures. MNE = Mean Normalized Expression.

Figures 7 and 8 show the transcriptional levels of 13 genes in liver of a total of 54 individual fish obtained from the low oxygen stress experiment. The fish were collected from six treatment groups, three that were fed high-energy diets and three that were fed low-energy diets. From each dietary group fish were either kept at normoxia (control), low oxygen or pair fed. 2-way ANOVA was used to search for effects of oxygen levels and dietary energy levels. The amount of energy in the feed had a stronger effect on the transcriptional levels than oxygen saturation levels. Significant effects of feed energy were observed for CuZn SOD (Figure 7A), Mn SOD (Figure 7B), CAT (Figure 7C), GR (Figure 7D), HSP70 (Figure 7E), HSP90A (Figure 7F), CYP1A (Figure 7H), and PSMC2 (Figure 8E). Significant effects of water oxygen saturation were observed for GR (Figure 7D), and IGFBP1B (Figure 8B). Interaction effects between feed energy content and water oxygen saturation were observed for GR (Figure 7D), and IGFBP1B (Figure 8B). Only two out of the 13 evaluated genes showed a significant effect of low oxygen exposure according to the RT-qPCR data by comparing the normoxia and low oxygen groups directly (high energy and low energy feed groups combined). Hypoxic condition resulted in lowered GR transcription (Figure 7D, t-test, P = 0.04, n = 18) and increased IGFBP1B transcription (Figure 8B, t-test, P = 0.0049, n = 18).

Correlation analyses of the individual RT-qPCR transcript data from the temperature and low oxygen stress experiments are shown in Additional file 5. From the temperature stress experiment, many of the evaluated target gene transcripts were strongly correlated with each other (Additional file 5, worksheet A). For example, the transcriptional level of HIF1A in these individuals was positively correlated with CuZn SOD, Mn SOD, GR, GPx1, MTOR, CYP1A, NDUFS1 and PSMC2 (Spearman’s rank-order correlation, R > 0.75). Many of the transcripts encoding oxidative stress markers were strongly correlated in fish exposed to heat stress.

To compare correlations of transcripts from the low oxygen exposure experiment, we combined the control normoxia fish from the high and low energy feeding groups (n = 18), and the low oxygen-exposed fish from the two dietary groups (n = 18), to search for altered correlations in fish kept at suboptimal oxygen saturation for 120 days (data shown in Additional file 5, worksheet B). HIF1A transcription was positively correlated to CuZn SOD and PSMC2 transcription in the low oxygen-exposed fish, but not in the control fish (Spearman’s rank-order correlation, R > 0.47). For most of the evaluated oxidative stress marker genes, low oxygen exposure induced few changed transcript correlations, except for Mn SOD that showed stronger correlations with NDUFS1 and PSMC2 in fish kept at low oxygen. NDUFS1, one of the genes selected from the cDNA libraries as a potential marker of both temperature and low oxygen stress, was positively correlated with IGFBP1A and PSMC2 in the low oxygen-exposed fish, but not in the control fish. Both HSP70 and HSP90A were positively correlated with IGFBP1B in the low oxygen-exposed fish, but not in the control fish. CYP1A, a much-studied biomarker that easily changes expression after environmental stress, showed several low oxygen-mediated correlations, including positive correlations with CuZn SOD, Mn SOD, CAT, MTOR and HSP70. Additional file 6 shows sequences in Fasta format of all assembled contigs from the four cDNA libraries (A-D) discussed in this work.

## Discussion

In this work we wanted to compare the transcriptional responses to chronic high temperature and low oxygen stress in Atlantic salmon to elucidate possible negative effects on farmed Atlantic salmon as a consequence of climate change. To do so we obtained samples from two independent experiments, one temperature stress experiment conducted in 2009 and described by Hevrøy et al. [30], and one low oxygen stress experiment conducted in 2011. Global transcriptional profiling data were obtained from four cDNA libraries sequenced with 454 FLX technology. IPA Upstream Regulator analysis aims to identify which transcriptional regulators that may be responsible for the observed change in a dataset, and was used for comparison of the two datasets. Based on the suggested upstream regulators the two stressors seem to affect many transcription factors regulating similar processes in liver cells. In line with established knowledge, at the molecular level these stressors appear to affect the rate of protein synthesis and lead to a metabolic rate suppression that ultimately results in reduced growth. This study thus suggests that both temperature stress and low oxygen induce metabolic depression in Atlantic salmon.

In general, many genes were significantly down-regulated in fish exposed to 19°C compared to the control fish kept at optimal condition at 13°C. This pattern was verified with the RT-qPCR analyses. Fish held at 15°C or above had reduced growth compared to the controls. In ectoderms, abiotic factors such as temperature determine the amount of energy spent on maintenance and growth, as most biological processes, i.e. protein synthesis and degradation, are temperature dependent. At temperatures above optimal reduced growth is inevitable [5], as recently shown in longtime elevated temperature experiments with Atlantic salmon [6, 30]. The current study shows that long-term exposure to sub-optimal oxygen saturation also has a negative effect on growth in Atlantic salmon. The growth effects of hypoxia are often explained in terms of loss of appetite and assimilation efficiency, and in terms of digestion [32]. A similar loss of appetite has been shown in large Atlantic salmon continuously exposed to 19°C [4]. At the cellular level, protein synthesis and ion-pumping through Na,K-ATPase represent key targets of hypoxia causing energy reallocation [33]. By comparing the two datasets, temperature and low oxygen stress seem to induce effects involving many similar mechanisms. However, surprisingly few of the top-ranked genes in the cDNA libraries were common between the two treatments. Only 19 contigs with similar annotation were identified as being affected both by temperature and low oxygen. This suggests that although chronic high temperature and low oxygen stress affects many identical mechanism in fish, they do not necessary invoke these effects through differential regulation of the same individual genes. The low degree of overlap may in part rely on the two different methods applied for cDNA library construction, as discussed below. In marine environments it is generally held that dissolved oxygen concentrations below 2-3 mg O_2_/L is considered hypoxic [17]. By this definition, the low oxygen stress used in the current experiment did not account as hypoxia, but for simplicity we use the “low oxygen stress” and “hypoxia” terms interchangeable throughout the discussion. In coldwater salmonids, behavioral responses to hypoxia have been detected already at 8 mg O_2_/L [34], but dissolved oxygen conditions below 6 mg O_2_/L is generally regarded as hypoxic for Atlantic salmon [19, 35]. Whether the observed responses reported here should be considered as stress or allostasis [36] is arguable, but animals experiencing long-term disturbances will tend to adjust their physiology toward normal homeostasis. Even so, over time these moderate stressors may have a negative impact on fitness, as suggested by the reduced growth seen in both experiments.

Interestingly, temperature stress appears to down-regulate several important liver transcription regulators. MYCN, HNF1A, HNF4A and NFE2L2 were among the transcription regulators that were inhibited by high temperature. This finding suggests that heat stress may have had an effect on the transcriptional rate in salmon liver. At the same time, most of the upstream regulators with a predicted activated state are typically associated with responses induced by chemical drugs. Thus, the key liver transcriptional factors affected by temperature stress suggest an effect on overall transcription, while at the same time the environmental stimuli seems to induce transcription of genes most often linked to effects of toxicants. The results suggest a switch toward increased transcription of protective enzymes at the cost of synthesis of maintenance enzymes. Based on the RT-qPCR results from the heat stress study, it appears clear that liver transcription in Atlantic salmon is considerable affected at temperatures above 17°C. Of the evaluated markers for oxidative stress, four out of five genes, CuZn SOD, Mn SOD, GPx1 and GR, were lower expressed in liver of fish kept at 19°C than in fish kept at optimal temperature at 13°C, while three genes, CuZn SOD, GPx1 and GR, also showed significant lower expression in fish kept at 17°C. This could be due to reduced mitochondrial ROS production as a result of reduced overall metabolism at higher temperature, although, in general, elevated environmental temperature results in enhanced oxygen consumption and ROS production and thereby increased oxidative stress in fish [37]. High temperature mediated lower expression of several of the other evaluated genes also, including HIF1A. The only gene that showed a significant higher expression in heat-stressed fish was HSP90B. In heat stressed fish, HSP90B was positively correlated with HSP70, but this transcript showed no significant correlation with any of the other evaluated genes, as opposed to for example HIF1A, whose expression was significantly correlated with all evaluated transcripts except for the two heat shock protein transcripts HSP70 and HSP90B.

Several overrepresented GO terms in fish exposed to low oxygen were associated with tissue development and growth. The GO enrichment analysis thus suggests a distinct response to low oxygen at the molecular level, with the sub-optimal oxygen concentration affecting transcripts encoding proteins important for continued growth. According to the IPA analysis, hypoxia induced effects on “organismal development” including lipid and nucleic acid metabolism at the molecular level, with protein ubiquitination as the most strongly affected pathway. The predicted top upstream regulators, 1,2-dithiol-3-thione, sirolimus, pirintrix acid, CD437 and 5-fluorouracil, suggest an effect leading to increased apoptosis and negative weight gain. Glutathione depletion and signaling effects possibly induced by nuclear factor (erythroid-derived 2)-like 2 (NFE2L2) in the liver seems a likely explanation for these findings. NFE2L2 is a transcription activator that binds to antioxidant response elements (ARE) in the promoter regions of target genes important for the coordinated regulation of genes in response to oxidative stress [38]. Of the oxidative stress marker genes evaluated with RT-qPCR, only GR showed a significant effect of low oxygen treatment. GR is crucial in glutathione metabolism and maintains high levels of reduced glutathione in the cytosol. In a previous study in which Atlantic cod (*Gadus morhua*) were exposed to 46% O_2_ saturation for six weeks, we observed down-regulation of transcripts encoding CuZn SOD and GPx3 [39]. Altered regulation of genes involved in glutathione metabolism strengthens the predicted effect of hypoxia on NFE2L2 regulated oxidative stress markers. Three of the predicted five top significant upstream regulators induced by hypoxia were also among the top five most significant upstream regulators induced by temperature stress, i.e. 5-fluorouracil, CD437 and sirolimus, suggesting a partly overlapping response to the two stressors.

A compelling finding was that among the 19 common genes were two transcripts encoding proteins typically involved in detoxification of persistent organic pollutants (POPs), i.e. CYP1A and GSTA1. Both transcripts were higher expressed in temperature-stressed fish liver. Due to the high fat content in muscle, farmed Atlantic salmon are prone to accumulate relatively high levels of lipophilic POPs in fillet and liver [40]. One can therefore speculate that elevated temperature may have affected the storage and turnover of POPs in salmon muscle and liver, as influx and efflux rates of toxicants across membranes increase with increasing temperature [29]. In temperature-stressed salmon, lipids stored in muscle tissue are increasingly being used for maintenance energy metabolism [30]. EROD activity is temperature dependent in fish [41, 42], so if increased EROD activity over time is followed by increased transcription, a temperature effect on CYP1A transcription might be expected. In gills of rainbow trout (*Oncorhynchus mykiss*) held at 8 or 23°C for two weeks, heat stress up-regulated several drug-metabolizing protein transcripts including phase I and II enzyme transcripts such as CYP1A, CYP1C1, UGT2B17, and xenobiotic transporter ABCG2 [43], clearly suggesting a temperature effect on drug-metabolizing enzyme transcription in salmonids. Since both aryl hydrocarbon receptor (AhR) and HIF-1 compete for aryl hydrocarbon nuclear translocator (ARNT), hypoxia could be expected to decrease the expression of P450 genes [44]. Indeed, in Atlantic cod exposed to 46% hypoxia for six weeks we observed CYP1A transcript down-regulation [39]. Rahman and Thomas [45] also observed a down-regulation of CYP1A mRNA and protein levels in liver of Atlantic croaker (*Micropogonias undulatus*) exposed to hypoxia (dissolved oxygen, DO: 1.7 mg/L for 2 to 4 weeks) compared to fish held in normoxic condition, and suggested that hypoxia-induced down-regulation of CYP1A is due to alterations of nitric oxide and oxidant status, and cellular IL-1beta and HIF-alpha levels. In threespine stickleback (*Gasterosteus aculeatus*) acutely exposed to hypoxia for 4-48 hours, Leveelahti et al. [46], however, observed increased expression of CYP1A2 mRNA, a finding also confirmed at the protein level by EROD activity measurement. These findings suggest that hypoxia exposure may affect the expression of AhR-mediated P450 genes. The reason for the altered transcription of CYP1A in hypoxia-stressed fish, which we were not able to independently verify with RT-qPCR in the current work, should be studied further.

Metabolic responses to ensure cell survival during hypoxia exposure involve metabolic reorganization to decrease ATP demands to match the reduced capacity for ATP production [47]. Several signal transduction cascades, including AMP-activated protein kinase (AMPK) and HIF-1, are activated in response to hypoxia in fishes and other vertebrates [48, 49]. AMPK activation in mammals inhibits energetically costly anabolic processes such as protein synthesis, glycogen synthesis, and fatty acid synthesis rates [47]. One AMPK gene, the 5-amp-activated protein kinase subunit beta-1 (PRKAB1), was found included in our dataset from the low oxygen exposure gene list but was not present in the normoxia library gene list. The PRKAB1 subunit of AMPK may be a positive regulator of AMPK activity [38]. Also present in the hypoxia gene list but not present in the normoxia gene list was the hypoxia-inducible factor 1A (HIF1A). HIF1A is a transcription factor that functions as a master regulator of gene expression in response to hypoxia [50]. HIF-1 protein is a heterodimer composed of an alpha and a beta subunit that is involved in cellular processes such as energy metabolism, apoptosis, proliferation, death and growth. Both acute and chronic hypoxia can distinctly affect mRNA levels of HIF-1, and this gene has been suggested as a reliable fish biomarker of hypoxia exposure [51]. Heat treatment mediated a reduced expression of HIF1A mRNA in liver of Atlantic salmon. According to the RT-qPCR data HIF1A transcription appeared to be stimulated at moderate heat stress (15°C), but was significantly lower at more severe heat stress (17-19°C). A similar response pattern has been observed in the North Sea eelpout (*Zoarces viviparous*), with elevated DNA binding activity of HIF-1 during mild heat exposure (18°C) but impaired activity at more severe heat stress [35]. A possible link between temperature and HIF-1 activity has previously also been shown for crucian carp (*Carassius carassius)*[52].

Insulin-like growth factors binding proteins (IGFBPs) play important roles in down-regulating IGF availability and cell growth and development in vertebrates exposed to hypoxic stress [53]. Gracey et al. [20] observed increased transcription of IGFBP1 in liver of longjaw mudsucker and shortjaw mudsucker (*Gillichthys seta*) after acute exposure to hypoxia, in line with our finding. In zebrafish embryos it has been shown that hypoxia strongly induces transcription of the IGFBP1 [54, 55]. Overexpression of IGFBP1 resulted in reduced growth in zebrafish embryos under normoxic condition, suggesting that the IGFBP1 protein plays an important role on fish growth during hypoxia and may even be an activator of the HIF-1 system. In line with our finding, Rahman and Thomas [53] found that chronic hypoxia exposure (2-4 weeks) caused significant increase in liver IGFBP1 mRNA in Atlantic croaker. IGFBP1 transcription thus appears to be a good biomarker for chronic hypoxia also in Atlantic salmon. Two IGFBP1 genes have been found in Atlantic salmon possibility due to whole genome duplication [56, 57], and these seem to be differentially regulated at the transcriptional level in liver after chronic low oxygen stress but not after temperature stress. Heat stress, induced by a temperature increase from 13°C to 17°C, appears to increase the transcription of both IGFBP1A (this study) and IGFBP1B [30] in Atlantic salmon. Chronic low oxygen stress mediated a significant change only for the IGFBP1B ortholog.

Both applied cDNA library construction methods represent a semi-quantitative measure of transcript abundance. SSH cDNA libraries are prone to false positives, whereas normalized cDNA libraries, by removing high-abundant rRNA transcripts, increase sequence coverage depth and transcript diversity across non-rRNA populations [58]. For this reason we have not attempted to compare the libraries quantitatively. By using pooled samples, and two different library construction methods, the RNA-seq data presented here should be considered indicative rather than exact quantitative measures of molecular effects of treatments that mitigate effects at the physiological level, i.e. reduced growth. As expected, fewer large contigs, contigs of at least 500 bp, were obtained by using SSH cDNA libraries than by using normalized cDNA libraries. With SSH technology, about 2000 large contigs were obtained from the heat stress experiment, whereas about 8000 large contigs were obtained from the normalized libraries. More equal numbers were obtained from the four cDNA libraries by selecting contigs consisting of more than 50 reads for the functional analyses. The selected strategy may have created a bias toward higher expressed genes, but represents a trade-off between confounding the pathway analysis by using too many input genes and lost strength by not including enough low-expressed genes.

Additionally, duplicated salmon co-orthologs may represent a problem in comparing the RNA-seq and RT-qPCR data. Co-orthologs with high sequence similarity may show differential expression but obtain similar best annotation. This phenomenon may explain the apparently contradictory finding for the CYP1A gene. According to the SSH library data, CYP1A was up-regulated by heat stress, while at the same time the RT-qPCR analysis suggested CYP1A to be down-regulated. Differential expression of orthologous HIF1A genes has recently been described in cyprinids, with one of the orthologs being more sensitive to oxygen tension [59]. At present, no information on orthologous HIF1A genes and their potential differential regulation are available for Atlantic salmon.

## Conclusions

This study suggests that environmental stress such as high temperature and low oxygen saturation, possibly becoming more widespread by global warming, may negatively affect growth in farmed Atlantic salmon. While heat stress in general appears to reduce the overall transcriptional rate, increased protein catabolism appears to be one of the main effects of low oxygen saturation stress. Predicted upstream transcriptional regulators suggest that the two types of stress affect many identical mechanisms in liver cells resulting in a metabolic depression.

## Methods

### Animal trial and experimental feeds

The temperature experiment was conducted at Matre Research Station, Institute of Marine Research, Matredal, Norway (61°N). Large immature Atlantic salmon (NLA strain) with a body mass of 1.6 ± 0.1 kg were randomly distributed into 12 3 m^2^ indoor tanks on August 6, 2009. After acclimation, on October 2, 2009, temperatures were adjusted to 13°C, 15°C, 17°C and 19°C, with triplicate tanks in 35 g/L seawater for each temperature and oxygen levels at 90% saturation (8 mg and 6 mg O_2_/L at 13 and 19°C, respectively). Temperatures were maintained at these levels until fish sampling on November 16 after 45 days of exposure. All fish were fed a commercial diet (Optiline, Skretting ARC, Stavanger, Norway). The feed contained 34.9% lipid, 37.9% protein, 5.8% ash, and 6.2% moisture, and had a gross energy content of 21.7 MJ/kg digestible energy (DE). At the end of the experiment, 36 fish, with nine fish from each treatment (three from each of the triplicate tanks with similar treatment) were collected 4 hours postprandial for weight and length measurements and tissue collection. The fish were killed with a blow to the head without sedation. Liver tissue samples for RNA extraction were immediately dissected out and flash frozen on liquid nitrogen, and stored at 80°C until further analysis. A detailed description of this experiment, including feeding and fish husbandry, is given in Hevrøy et al. [30].

The low oxygen experiment was conducted at Lerang Research Station, Skretting AS, Lerang, Norway (59 °N) between April and August, 2011. Immature Atlantic salmon of NLA strain weighing between 1.5-2.0 kg were distributed into 3 m^2^ tanks on April 11, 2011. All fish were pit tagged prior to the experiment. Using triplicate tanks for each treatment, Atlantic salmon were divided into 18 tanks and given six different treatments. Half of the fish were fed a high-energy diet (22 MJ DE, 9 tanks), and the other half a low-energy diet where lipids were exchanged with carbohydrate (20.5 MJ DE, 9 tanks). The high-energy diet contained 36.9% lipid, 38.7% protein, 5.0% ash and 5.5% moisture, while the low-energy diet contained 31.6% lipid, 36.4% protein, 5.0% ash and 6.5% moisture. One group of fish was fed a diet containing the same energy level as fish kept at low oxygen, called pair feeding to discriminate feed intake effects. The fish were presented with the following three different treatments; optimum oxygen (7-8 mg O_2_/L), optimum oxygen with pair feeding (7-8 mg O_2_/L), or low oxygen (4-5 mg O_2_/L) all in triplicate tanks (n = 9, N = 54). Low oxygen levels were simulating typical natural farming conditions [60], with cyclic low dissolved oxygen levels during night and increased normal levels during day with average 6 mg O_2_/L between 12:00-18:00 h. Temperature was kept constant at 12°C during the trial with stable 35 g/L seawater and the fish were reared under a simulated natural photoperiod. In total 54 fish were sampled 4 hours postprandial after four months of treatment on August 23, 2011. Liver tissue samples for RNA extraction were immediately dissected out and flash frozen on liquid nitrogen, and stored at 80°C until further analysis. The experiment complied with the guidelines of the Norwegian Regulation on Animal Experimentation and EC Directive 86/609/EEC, and the National Animal Research Authority approved the protocol.

### Biological performance data

During the experiments, daily feed intake was monitored to secure optimal growth recordings (detailed description given in Hevrøy et al. [30]; Vibeke Vikeså, unpublished results). All fish were recorded for weight and fork length to the nearest g and nearest 0.5 cm at the start and at end of the experiments (N = 538 and N = 990, temperature and low DO trial). To obtain comparable relation in somatic growth measurements, thermal growth coefficients were determined. The thermal growth coefficient (TGC) was calculated as TGC = ((W_2_^0.333^ - W_1_^0.333^)/(Σ C°)) × 1000, where W_1_ and W_2_ are initial and final body mass in grams and Σ C° are sum day-degree in the experiment.

### RNA isolation

Liver tissues from the Atlantic salmon were thoroughly homogenized before RNA extraction using a Precellys 24 homogenizer by ceramic beads CK28 (Bertin Technologies, Montigny-le-Bretonneux, France). Total RNA was extracted using the BioRobot EZ1 and RNA Tissue Mini Kit (Qiagen, Hilden, Germany) and treated with DNase according to the manufacturer's instructions and eluted in 50 μL RNase-free MilliQ H_2_O. The RNA was then stored at -80°C before further processing. RNA quality and integrity were assessed with the NanoDrop ND-1000 UV-Vis Spectrophotometer (NanoDrop Technologies, Wilmington, DE, USA) and the Agilent 2100 Bioanalyzer (Agilent Technologies, Palo Alto, CA, USA). The RNA 6000 Nano LabChip kit (Agilent Technologies, Palo Alto, CA, USA) was used to evaluate the RNA integrity of the liver samples. The 260/280 and 260/230 nm ratios of the extracted RNA were 2.1 ± 0.0 and 2.1 ± 0.0, respectively (mean ± SD). The RNA integrity numbers (RIN) of the liver samples used for RT-qPCR from the temperature stress and hypoxia cDNA libraries were 9.6 ± 0.1 (n = 36) and 8.8 ± 0.3 (n = 54) (mean ± SD), respectively.

### Suppressive subtractive hybridization (SSH) and normalized cDNA library construction

Pooled RNA from liver of Atlantic salmon from four treatment groups (13°C versus 19°C, and normoxia versus low O_2_) was used to construct cDNA libraries for sequencing. From the heat stress experiment, we pooled RNA from six fish from the control group and six fish from the high temperature group for construction of two suppressive subtractive hybridization (SSH) cDNA libraries. Pooled RNA, obtained from nine individuals from the normoxia and nine individuals from low oxygen experimental groups fed high-energy diets, was used to create the normalized cDNA libraries.

SSH was performed using the Clontech PCR Select cDNA Subtraction Kit (Clontech, Mountain View, CA) following the manufacturer’s recommendations. cDNA subtraction was performed in both directions. Forward subtracted libraries were designed to be enriched for genes that were up-regulated in liver of Atlantic salmon by heat stress (19°C), and reverse subtracted libraries were designed to be enriched for genes that were down-regulated by heat stress. Pooled mRNA samples from liver of fish exposed to 19°C were used as testers in the forward subtractions and as drivers in the reverse subtractions. Pooled mRNA samples from liver of fish held at 13°C were used as drivers in the forward subtractions and as testers in the reverse subtractions. To evaluate subtraction efficiency, the abundance of transcripts of the housekeeping gene *ubiquitin* was examined by PCR. For SSH cDNA libraries, mRNA from each sample was isolated using the NucleoTrap mRNA Mini Kit (Macherey-Nagel, Düren, Germany). The Agilent Bioanalyzer with the RNA 6000 Nano LabChip kit and the DNA 7500 Kit (Agilent Technologies, Waldbronn, Germany) was used to evaluate the quality of the mRNA and cDNA samples used for cDNA library construction. 200 ng of mRNA from each sample was used for cDNA synthesis according to the GS FLX Titanium Rapid Library Preparation Kit (Roche Applied Sciences, Basel, Switzerland).

For normalized cDNA library construction, mRNA was purified from 10 μg total RNA by exonuclease digestion followed by LiCl precipitation (mRNA-Only Eucaryotic mRNA Isolation Kit, Epicentre, Madison, WI, USA). 1 μg mRNA was used for first-strand cDNA synthesis. cDNA synthesis and amplification was done according to the Mint-Universal cDNA Synthesis Kit user manual (Evrogen, Moscow, Russia). 800 ng amplified cDNA was used as starting material in the normalization reaction using the Trimmer Kit (Evrogen, Moscow, Russia). Normalized material was re-amplified for 18 cycles. 2 μg of normalized cDNA was digested with 10 Units SfiI for 2 hours at 48°C. Fragments larger than 800 bp were isolated from a LMP Agarose Gel and purified using the MinElute Gel Extraction Kit (Qiagen, Hilden, Germany). 200 ng purified cDNA fragments were ligated to 100 ng Sfi cut and dephosphorylated pDNR-lib Vector (Clontech) in 10 μL volume using the Fast Ligation Kit (NEB, Ipswich, MA, USA). Ligations were desalted by ethanol precipitation, and re-dissolved in 10 μL water. 3 times 1.5 μL desalted ligation was used to transform NEB10b competent cells (NEB, Ipswich, MA, USA). 96 clones were randomly chosen for Sanger sequencing to verify successful normalization. For each library roughly 2 million clones were plated on LB-Cm plates, scrapped off the plates and stored as glycerol stocks at -70°C. One half of the cells were used to inoculate a 300 ml Terrific Broth/Cm culture, which was grown for 5 hours at 30°C. Plasmid DNA was prepared using standard methods (Qiagen, Hilden, Germany). 200 μg of purified plasmid DNA was digested with 100 Units SfiI for 2 hours at 48°C. cDNA Inserts were gel purified (LMP-Agarose/MinElute Gel Extraction Kit) and ligated to high-molecular-weight DNA using a proprietary Sfi-linker. Library generation for the 454 FLX sequencing was carried out according to the manufacturer’s standard protocols (Roche/454 life sciences, Branford, CT 06405, USA).

### 454 FLX sequencing

Atlantic salmon liver tissue cDNA libraries from the temperature stress trial were prepared as stated above and sequenced according to the Roche 454 GS FLX protocol using titanium chemistry (Roche 454 Life Sciences, Branford, CT, USA) at the Ultra-high Throughput Sequencing Platform of the Centre for Ecological and Evolutionary Synthesis (CEES), Department of Biology, University of Oslo, Norway. 454 FLX sequencing, data processing and data assembly of the normalized liver cDNA libraries were carried out by LGC Genomics GmbH, Berlin, Germany. Nucleotide sequences were incorporated into quality filtered flowgram (SFF) files using the 454’s software and applied in downstream analyses. Library generation for the 454 FLX sequencing of the samples was carried out according to the manufacturers standard protocols (Roche 454 Life Sciences, Branford, CT, USA). Briefly, the concatenated inserts were sheared randomly by nebulization to fragments ranging in size from 400 to 900 bp. These fragments were end polished and the 454 A and B adaptors that are required for the emulsion PCR and sequencing were added to the ends of the fragments by ligation. The resulting fragment library was sequenced on 3 individual 1/4 picotiter plates (PTP) on the GS FLX using the Roche 454 titanium chemistry.

### Clustering, assembly and read processing

As a quality measure in search for possible microbial contamination, i.e. impurities in the nucleotides under investigation, all reads generated by the FLX sequencer were subjected to taxonomic profiling using MEtaGenome ANalyzer (MEGAN, version 3.9.) using default settings [61]. Reads longer than 50 nt were aligned to the GenBank non-redundant protein database (Blastx) [62] using a cut-off e-value of 1e-6, and the Blast results used as input in the MEGAN analyses.

Prior to assembly the sequence reads were screened for the Sfi-linker that was used for concatenation, the linker sequences were clipped out of the reads and the clipped reads assembled to individual transcripts using the Newbler software version 2.6 at default settings (build: 20110517_1502). SFF files were assembled using the Newbler software with minimum overlap 80 bp and minimum identity 96% (“-ml 80 -mi 96 -cdna -ace").

### Functional analyses

Four sets of assembled “isotigs” (contigs) were used in the downstream functional analyses from the cDNA libraries. For gene ontology (GO) and Ingenuity Pathway Analysis (IPA) analyses (Ingenuity Systems, Inc., Redwood City, CA, USA), all isotigs consisting of 50 or more reads were used. Blast2GO [63] was used to annotate and analyze all isotigs consisting of 50 reads or more in the four SSH cDNA library assemblies. Blast2GO analyses were run using a cut-off e-value of 1e-3 (Blastx) and 1e-6 (mapping). GO enrichment analyses were performed with Fisher's exact test applying the GOSSIP tool [64] as integrated in the Blast2GO software.

Gene lists from the four cDNA libraries as annotated with Blast2GO were used for IPA pathway analysis. Since IPA only can map mammalian homolog identifiers, GeneCards IDs were submitted for biological function and pathway analysis, using top Blastx hits and assuming orthologous genes have the same function. A limited number of fish-specific genes with no mammalian homologs were for this reason not included in the pathway analysis. IPA could map and identify 428 (19°C), 639 (13°C), 1281 (normoxia) and 1341 (low O_2_) differently expressed genes from the four gene lists, to be included in pathway analyses.

### Quantitative real-time RT-qPCR

PCR primer sequences used for quantification of the transcriptional levels of selected genes, as well as the reference genes, are shown in Table 3. In total 17 genes were quantified with RT-qPCR, of which 3 were selected as potential reference genes. Blastx or Blastn was used to determine PCR assay specificity. The reaction specificity of each assay was checked by examining the melting curves generated with a dissociation protocol from 65 to 97°C.

**Table 3:** **PCR assays, including primers sequences, accession numbers, amplicon sizes and PCR efficiencies**

Gene	Gene product	Accession no.	Forward primer	Reverse primer	Amplicon size (bp)	PCR efficiency*
CuZn SOD	CuZn superoxide dismutase	BG936553	CCACGTCCATGCCTTTGG	TCAGCTGCTGCAGTCACGTT	140	1.92/2.02
Mn SOD	Mn superoxide dismutase	DY718412	GTTTCTCTCCAGCCTGCTCTAAG	CCGCTCTCCTTGTCGAAGC	209	1.85/1.88
CAT	Catalase	BG935638	GGGCAACTGGGACCTTACTG	GCATGGCGTCCCTGATAAA	59	1.85/2.12
GPX1	Glutathione peroxidase 1	EH033571	TCTCCTGCCATAACGCTTGA	GTGATGAGCCCATGGCCTTA	137	1.84/-
GR	Glutathione reductase	BG934480	CCAGTGATGGCTTTTTTGAACTT	CCGGCCCCCACTATGAC	61	2.00/1.91
HSP70	Heat shock protein 70	BG933934	CCCCTGTCCCTGGGTATTG	CACCAGGCTGGTTGTCTGAGT	121	-/1.90
HIF1A	Hypoxia-inducible factor 1A	DY708816	CCACCTCATGAAGACCCATCA	TCTCCACCCACACAAAGCCT	101	2.20/2.26
IGFBP1A	Insulin-like growth factor binding protein 1A	KC122927	GGTCCCTGTCATGTGGAGTT	TTCCAGAAGGACACACACCA	184	2.10/2.08
IGFBP1B	Insulin-like growth factor binding protein 1B	AY662657	GAGGACCAGGGACAAGAGAAAGT	GCACCCTCATTTTTGGTGTCA	101	-/2.02
MTOR	Mechanistic target of rapamycin (serine/threonine kinase)	BT072258	CAGCCTGAGGCCCTGAATAA	CTCCACTTGGGTTGGCACAT	114	1.97/1.95
CYP1A	Cytochrome P450, family 1, subfamily A	>contig00118 length = 2495 numreads = 57	ATC GGACGCAACGAGGTCTA	TGACAGCGCTTGTGCTTCAT	128	1.97/2.02
NDUFS1	NADH dehydrogenase (ubiquinone) Fe-S protein 1, 75 kDa	>contig00384 length = 2136 numreads = 57	TGCTGCAGGACATCGCTAAC	TGGTTTGCACAGAGCTCAAGA	135	1.94/2.01
PSMC2	Proteasome (prosome, macropain) 26S subunit, ATPase, 2	>contig01910 length = 1544 numreads = 106	ATCAGGGTCATCGGCTCAGA	GCCCCTCCAATAGCGTCAAT	132	1.94/2.02
HSP90B	Heat shock protein 90B	>contig03769 length = 1183 numreads = 111	CCACCATGGGCTACATGATG	CCTTCACCGCCTTGTCATTC	114	1.97/1.95
EEF1AB	Eukaryotic translation elongation factor 1AB (refgen)	AF321836	CCCCTCCAGGACGTTTACAAA	CACACGGCCCACAGGTACA	57	1.99/2.01
ACTB	Beta-actin (refgen)	BG933897	CCAAAGCCAACAGGGAGAA	AGGGACAACACTGCCTGGAT	102	2.06/1.90
RPL13	Ribosomal protein L13 (refgen)	NM_001141291	CCAATGTACAGCGCCTGAAA	CGTGGCCATCTTGAGTTCCT	110	-/1.91

*Temperature experiment/low O_2_ experiment.

RT-qPCR was conducted as previously described by Olsvik et al. [65]. Briefly, a two-step real-time RT-PCR protocol was used to quantify the transcriptional levels of the selected genes. The RT reactions were run in duplicate on a 96-well reaction plate with the GeneAmp PCR 9700 machine (Applied Biosystems, Foster City, CA, USA) using TaqMan Reverse Transcription Reagent containing Multiscribe Reverse Transcriptase (50 U μL^-^) (Applied Biosystems, Foster City, CA, USA). Two-fold serial dilutions of total RNA were made for efficiency calculations. Six serial dilutions (1000–31 ng RNA) in triplicates were analyzed in separate sample wells. Total RNA input was 500 ng in each reaction for all genes. No template controls (ntc) and RT-controls were run for quality assessment for each PCR assay.

Reverse transcription was performed at 48°C for 60 min by using oligo dT primers (2.5 μM) for all genes in 50 μL total volume. The final concentration of the other chemicals in each RT reaction was: MgCl_2_ (5.5 mM), dNTP (500 mM of each), 10X TaqMan RT buffer (1X), RNase inhibitor (0.4 U μL^-^) and Multiscribe reverse transcriptase (1.67 U μL^-^) (Applied Biosystems). Twofold diluted cDNA (2.0 μL cDNA in each RT reaction) was transferred to 384-well reaction plates and the qPCR run in 10 μL reactions on the LightCycler 480 Real-Time PCR System (Roche Applied Sciences, Basel, Switzerland). Real-time PCR was performed using SYBR Green Master Mix (LightCycler 480 SYBR Green master mix kit, Roche Applied Sciences, Basel, Switzerland), which contains FastStart DNA polymerase, and gene-specific primers (500 nM of each). PCR was achieved with a 5 min activation and denaturizing step at 95°C, followed by 45 cycles of a 10 s denaturing step at 95°C, a 20 s annealing step at 60°C and a 30 s synthesis step at 72°C. Target gene mean normalized expression (MNE) was determined using a normalization factor based upon ACTB and EEF1AB for the temperature exposure data and ACTB, EF1AB and RPL13 for the low oxygen exposure data, as calculated by the geNorm software [66]. All these transcripts were stably expressed among the 30 evaluated samples, with *geNorm* stability scores of M < 0.28.

### Statistics

Significant differences among treatments were assessed with t-test, 1-way analysis of variance (ANOVA) (temperature stress experiment) or 2-way ANOVA (low O_2_ experiment). *Post hoc* testing of significant differences was assessed by using the Tukey's HSD test. T-test was used to search for hypoxia effects from the low oxygen experiment by combining data from the high-energy and low-energy feed groups. In case of significantly different standard deviations as determined by the Bartlett’s test, the data was log transformed before ANOVA analysis. Outliers were detected by using the ROUT method [67]. The GraphPad Prism 5.0 software (GraphPad Software, Inc., San Diego, CA, USA) was used for statistical analyses of the transcriptional data. Correlation analysis was performed using the program Statistica 8.0,(Statsoft Inc., Tulsa, USA). Contigs and isotigs were annotated with the Blast2GO software. The functional pathway analyses were generated through the use of IPA (Ingenuity Systems, http://www.ingenuity.com). A significance level of P < 0.05 was used for all tests.

### Availability of supporting data

All supporting data are included as additional files.

## Electronic supplementary material

Additional file 1: **Gene transcripts up- (worksheet A) and down-regulated (worksheet B) by temperature treatment in liver of Atlantic salmon.** Based on data obtained from two SSH cDNA libraries and sorted by the number of reads. Only contigs consisting of 50 or more reads were included in IPA pathway analysis. (XLSX 139 KB)

Additional file 2: **Most abundant gene transcripts in liver of Atlantic salmon A) kept at normal oxygen saturation level or B) exposed to low oxygen saturation stress.** Based on data obtained from two normalized cDNA libraries and sorted by contig length. Only contigs consisting of 50 or more reads were included in IPA pathway analysis. (XLSX 431 KB)

Additional file 3: **A) Enriched gene ontologies (GOs) in liver of Atlantic salmon exposed to 19°C. B) Enriched GOs in liver of Atlantic salmon exposed to low oxygen saturation stress (>50 reads), C) Enriched GOs in liver of Atlantic salmon exposed to low oxygen levels (all reads).** Duplicates were removed. GO enrichment analysis was conducted by using the Fisher’s Exact Test as implemented in the Blast2GO software. P < 0.05. (XLSX 482 KB)

Additional file 4: **Possible upstream regulators in liver of Atlantic salmon exposed to temperature stress as suggested by IPA analysis.** (XLS 76 KB)

Additional file 5: **Correlation analysis of evaluated transcripts in liver of Atlantic salmon exposed to A) temperature stress and B) low oxygen saturation stress.** (XLSX 75 KB)

Additional file 6: **Fasta sequences of assembled contigs consisting of more than 50 reads from the four cDNA libraries.** (XLSX 2 MB)
